# Vitamin D in the Context of Evolution

**DOI:** 10.3390/nu14153018

**Published:** 2022-07-22

**Authors:** Carsten Carlberg

**Affiliations:** 1 Institute of Animal Reproduction and Food Research, Polish Academy of Sciences, 10-748 Olsztyn, Poland; c.carlberg@pan.olsztyn.pl; 2 School of Medicine, Institute of Biomedicine, University of Eastern Finland, 70211 Kuopio, Finland

**Keywords:** vitamin D, evolution, energy metabolism, immune system, calcium homeostasis, migration of *Homo sapiens*

## Abstract

For at least 1.2 billion years, eukaryotes have been able to synthesize sterols and, therefore, can produce vitamin D when exposed to UV-B. Vitamin D endocrinology was established some 550 million years ago in animals, when the high-affinity nuclear receptor VDR (vitamin D receptor), transport proteins and enzymes for vitamin D metabolism evolved. This enabled vitamin D to regulate, via its target genes, physiological process, the first of which were detoxification and energy metabolism. In this way, vitamin D was enabled to modulate the energy-consuming processes of the innate immune system in its fight against microbes. In the evolving adaptive immune system, vitamin D started to act as a negative regulator of growth, which prevents overboarding reactions of T cells in the context of autoimmune diseases. When, some 400 million years ago, species left the ocean and were exposed to gravitation, vitamin D endocrinology took over the additional role as a major regulator of calcium homeostasis, being important for a stable skeleton. *Homo sapiens* evolved approximately 300,000 years ago in East Africa and had adapted vitamin D endocrinology to the intensive exposure of the equatorial sun. However, when some 75,000 years ago, when anatomically modern humans started to populate all continents, they also reached regions with seasonally low or no UV-B, i.e., and under these conditions vitamin D became a vitamin.

## 1. Introduction

Evolution is a dominant driver of the development of biological processes and their adaption to changes in the environment. The statement “Nothing in biology makes sense except in the light of evolution” [1] underlines that evolution is an essential component for understanding the mechanisms of these processes. Accordingly, this review will discuss vitamin D, its nuclear receptor (NR) VDR and their molecular action in the light of evolution.

It was exactly 100 years ago that vitamin D was named a vitamin, because it is able to cure experimentally induced rickets in dogs and rats [2]. Since rickets is a bone malformation disorder in children, this and many other studies linked vitamin D to calcium homeostasis and bone remodeling [3]. However, calcium homeostasis is only one of multiple biological processes being regulated by vitamin D, such as detoxification, energy metabolism as well as innate and adaptive immunity [4]. In fact, the relationship between vitamin D and bone remodeling developed as one of the most recent evolutionary functions of vitamin D.

Furthermore, the consequences of the migration of modern humans from equatorial East Africa to regions of higher latitude will be reflected in relation to vitamin D’s possible role in skin lightening, particularly in European populations [5].

## 2. Sterols and Vitamin D Synthesis

**Figure 1:**
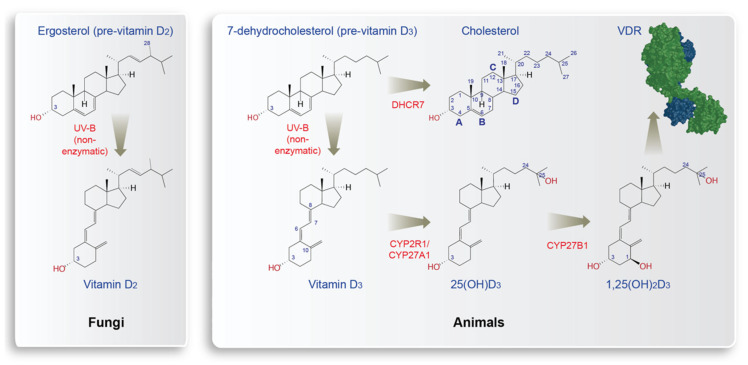
Principles of endogenous vitamin D_2_ and vitamin D_3_ production. In fungi, vitamin D_2_ is produced non-enzymatically when the sterol ergosterol is exposed to UV-B radiation. In cholesterol synthesizing animals (as well as in some plants, such as phytoplankton), 7-dehydrocholesterol reacts to vitamin D_3_ using the energy of UV-B. Animals express enzymes that convert vitamin D_3_ first to 25(OH)D_3_ and then to 1,25(OH)_2_D_3_. In animals (but not in fungi), vitamin D endocrinology developed and 1,25(OH)_2_D_3_ acts as a hormone binding with high affinity to the nuclear receptor VDR (green, the co-receptor RXR is shown in blue). In the example of cholesterol, the numbering of rings (A-D) and carbons (1-27) is indicated, while only key carbons are marked in the other molecules.

**Figure 2:**
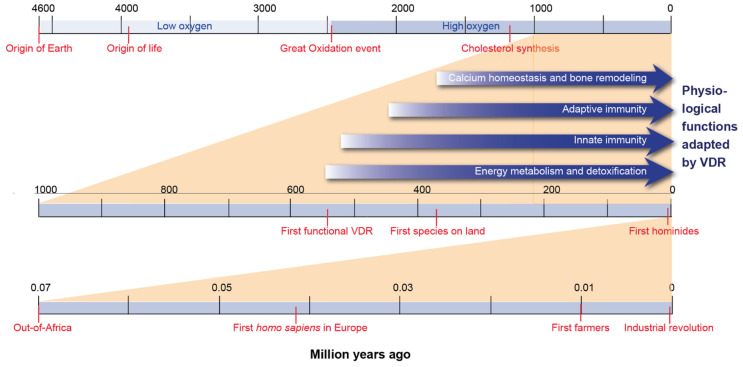
Timeline of evolution. The evolutionary history of the past 4.6 billion years (**top**), 1 billion years (**center**) and 70,000 years (**bottom**) are depicted. Important events discussed in this review are indicated and the time of their approximate occurrence is marked.

Sterols are lipophilic molecules carrying a four-ring skeleton, a hydroxyl group at carbon (C) 3 and a flexible side chain at C17 (Figure 1). The use of sterols in cell membranes is a characteristic difference between eu- and prokaryotes [6]. Some 2.45 billion years ago, atmospheric molecular oxygen (O_2_) concentrations drastically rose in the so-called great oxidation event [7] (Figure 2). This stimulated the development of complex eukaryotes with the help of new enzymes and biochemical pathways [8]. A key example is the biosynthesis of sterols, where four different types of enzymes require O_2_. Moreover, aerobic metabolism, such as oxidative phosphorylation, significantly increases the generation of energy from nutrients. In parallel, some of these new enzymes and pathways had the primary role of protecting from oxygen toxicity, which also may have been the primary role of sterols [9]. Thus, the occurrence of oxygen and sterols are tangled; without oxygen there is no sterol synthesis and many sterols protect from damage created by O_2_ and reactive oxygen species. Furthermore, some sterols can be considered as oxygen sensors [10].

There is a large number of naturally occurring sterols and species can be phylogenetically distinguished by their sterol profile. Sterols are primarily distinguished by the modification at C24 in their side chain. In animals, 24-desmethylsterols such as cholesterol (no additional group, 27 carbon atoms in total) are typical, while fungi have 24-demethylsterols, such as ergosterol (28 carbon atoms) (Figure 1). In contrast, plants produce a wide range of more than 250 different sterols, the most common of which are sitosterol, campesterol and stigmasterol [11]. Interestingly, cholesterol represents 1–2% of the plant sterol content, i.e., cholesterol is not unique to animals but can also be produced at least by some plants, such as algae [12]. Cholesterol is not only critical for membrane fluidity but also an important precursor for bile acids and steroid hormones [13]. Most eukaryotic species, including humans, can synthesize sterols de novo, but some others, such as insects, depend on a supply of sterols via their diet [14].

When sterols like ergosterol (pre-vitamin D_2_) in fungi or the direct cholesterol precursor 7-dehydrocholesterol (pre-vitamin D_3_) in animals and phytoplankton are exposed to UV-B radiation of 280–315 nm, they transform non-enzymatically to vitamin D_2_ and vitamin D_3_, respectively (Figure 1). First, the double bond between C7 and C8 of both types of sterols absorbs the energy of the radiation, which creates thermodynamically unstable pre-vitamin D molecules, in which their B ring is opened between C9 and C10, creating secosteroids [15]. Then elevated temperature catalyzes the isomerization of pre-vitamin D into vitamin D. Since both reactions do not require any enzyme, it is likely that vitamin D_2_ and vitamin D_3_ are evolutionary very old molecules that occurred as early as the ergosterol and cholesterol biosynthesis pathways evolved some 1.2 billion years ago (Figure 2). For example, for at least 750 million years, phytoplankton has produced vitamin D_3_ [16]. However, in early evolution, vitamins D_2_ and vitamin D_3_ may have been primarily side products of sterol biosynthesis in UV-B exposure, i.e., they had no signaling function since a respective endocrine system (see Section 3) had not evolved. Interestingly, continuous UV-B exposure can convert pre-vitamin D_3_ into lumisterol, tachysterol and other photoproducts [17], i.e., vitamin D_3_ precursors and metabolites are able to perform UV scavenging by rearranging double bonds within the molecule [18]. Thus, the pre-endocrine function of vitamin D was and still is the protection of DNA and proteins from mutagenesis and degradation, respectively.

Interestingly, the accumulation of vitamin D_3_ in the marine food chain [12,19] explains why salmon, as well as the liver of cod (“cod liver oil”), have a high content of the vitamins [20,21].

## 3. Evolution of Vitamin D Endocrinology

The core protein of an endocrine system is its receptor. A high-affinity receptor for vitamin D, the transcription factor VDR evolved some 550 million years ago [22]. However, contrary to its name, VDR is activated neither by vitamin D_2_ nor by vitamin D_3_ [23]. Carrying only one hydroxy group, both secosteroids are not polar enough to bind VDR. In fact, two hydroxylation reactions are required to form with 1α,25-dihydroxyvitamin D_3_ (1,25(OH)_2_D_3_) a vitamin D metabolite that offers three hydroxy groups for specific high-affinity binding to the ligand-binding domain (LBD) of VDR (Figure 1). This implies that the 25-hydroxylases cytochrome P450 (CYP) 2R1 and CYP27A1, as well as the 1α-hydroxylase CYP27B1, are key components of vitamin D endocrinology. They transform vitamin D_3_ into 25-hydroxyvitamin D_3_ (25(OH)D_3_) and 25(OH)D_3_ into 1,25(OH)_2_D_3_, respectively. Furthermore, as described for other hormones, the levels of 1,25(OH)_2_D_3_ need to be tightly regulated. This happens via the 24-hydroxylase CYP24A1 that converts 1,25(OH)_2_D_3_ to 1,24,25(OH)_3_D_3_ and inactivates the VDR ligand in this way [24]. Despite their hydroxyl groups, all vitamin D metabolites are lipophilic and need to be carried in hydrophilic serum and cellular liquids by transport proteins. Thus, for a functional endocrine system, specific receptor(s), metabolizing enzymes and transport proteins need to evolve [25].

VDR belongs to the transcription factor family of NRs, which in humans is formed by 48 genes [26]. Comparative genomics demonstrates that the closest relatives to VDR are the NR1I subfamily members pregnane X receptor (PXR) and constitutive androstane receptor (CAR), and the NR1H subfamily members liver X receptor (LXR) α and b, as well as the farnesoid X receptor (FXR) [27]. This indicates that VDR and its five relatives have a common ancestor and that the individual receptor genes developed by whole genome duplications in early vertebrate evolution [28]. Interestingly, the six NRs function as sensors for cholesterol derivatives, such as 1,25(OH)_2_D_3_, oxysterols and bile acids [29]. Moreover, FXR, VDR, CAR and PXR detect toxic secondary bile acids, such as lithocholic acid, and get activated by them [30,31,32,33]. This suggests that the prime function of the common ancestor of NR1H- and NR1I-type NRs was to act as a bile acid sensor. Accordingly, one of the first functions of VDR and its relatives was the regulation of genes encoding for enzymes of marine biotoxin degradation [4,34].

Detoxification reactions represent a specialized form of metabolism that allows a response to environmental conditions, such as the rise in toxic compounds. However, the most dominant environmental challenge of species is their diet, which is primarily composed of macro- and micronutrients. This created an evolutionary pressure, with the push of which the sensing of the levels of nutritional molecules like fatty acids, cholesterol and vitamins became the main function of NRs, such as peroxisome proliferator-activated receptors (PPARs), LXRs, retinoid acid receptors (RARs) and VDR [35,36]. This function is closely linked to the control of energy metabolism, which was and still is a prime task of many NRs, including VDR [37]. Accordingly, a significant proportion of the hundreds of VDR targets are metabolic genes [38,39,40,41].

Archetypical NRs were orphan receptors, as some members of the NR superfamily still are [42]. Comparative genomics suggests that in a stepwise evolutionary adaption, orphan, NRs changed critical amino acids within their LBD, so that a ligand-binding pocket got accessible to potential small lipophilic ligands. The 40 or more amino acids forming this pocket are specifically adapted to the shape and polarity of the ligand. Some 550 million years ago, this evolutionary adaptation process resulted in the first known VDR that binds 1,25(OH)_2_D_3_ at sub-nanomolar concentrations was found in the early jawless vertebrate sea lamprey (*Petromyzon marinus*) [22], meaning that VDR had evolved into a classical endocrine receptor, such as those for the steroid hormones estrogen, testosterone and progesterone. Crystal structure analysis of lamprey’s VDR ligand-binding domain [43] confirmed similar binding of 1,25(OH)_2_D_3_ as identified for human VDR [44]. In vertebrate evolution, amphibians, reptiles, bony fish, birds and mammals also learned to express functional VDR proteins [45]. Most species have only one *VDR* gene, but the genome of teleost fishes underwent a third whole genome duplication and contains even two *VDR* genes [46].

Since the levels of 1,25(OH)_2_D_3_ in lamprey are similar to that in higher vertebrates, respective enzymes, such as CYP2R1 and CYP27B1, must have co-evolved with VDR [22]. Similar co-evolution also happened for the vitamin D transport protein vitamin D binding protein (encoded by the *GC* gene) [25]. This indicates that some 150 million years before the first species left the ocean and had the need for a stable skeleton, vitamin D endocrinology was already established. Thus, from an evolutionary perspective, the control of calcium homeostasis was rather a secondary than a primary goal for establishing the vitamin D endocrine system.

## 4. Evolution of the Physiological Functions of Vitamin D

Possible harming invaders created since the early times of life on Earth a strong evolutionary pressure for developing defense mechanisms, such as an immune system. The innate immune system is evolutionarily older and found already in many non-vertebrate species, such as insects. It involves a number of barriers, such as skin and mucosa, and uses a limited set of pattern recognition receptors that detect only general features of possible pathogens. In contrast, the adaptive immune system developed some 500 million years ago in ectothermic cartilage fishes and uses antigen receptors, such as B and T cell receptors, that have a very high affinity and specificity to their antigens [47,48].

The growth of immune cells and their function in defense and tissue repair takes significant amounts of energy [49]. Key vitamin D target genes in this context are *PFKFB4* (6-phosphofructo-2-kinase/fructose-2,6-biphosphatase 4) in dendritic cells [50] and *FBP1* (fructose-bisphosphatase 1) in monocytes [51]. Therefore, the regulatory function of vitamin D and its receptor on energy metabolism were essential during the development of the immune system (Figure 2). Moreover, vitamin D modulates innate immunity through further target genes, such as those encoding for the antimicrobial peptide CAMP (cathelicidin) [52] and the toll-like receptor 4 co-receptor CD14 [51] in monocytes. Furthermore, in dendritic cells, which present antigens to T cells of the adaptive immune system, many genes respond to vitamin D [53]. In this way, vitamin D was and still is involved in efficient responses to pathogens, such as the intracellular bacterium *Mycobacterium tuberculosis* [54]. Moreover, the cluster of *HLA* (human leukocyte antigen) genes on human chromosome 6, many of which are vitamin D targets [41], is a “hotspot” of vitamin D-induced chromatin accessibility [55]. Thus, most non-skeletal functions of vitamin D, like the modulation of the immune system, developed before its regulation of calcium homeostasis and bone remodeling had been established (Figure 2).

Some 385 million years ago, the next important step in vertebrate evolution happened: some species moved from the ocean onto land and had to develop a skeleton supporting locomotion under gravitational forces [25] (Figure 2). At earlier times, calcified cartilage and dermal bone had already been developed by cartilage fishes like sharks. Bone fishes even had replaced this cartilage with bone [56]. In the calcium-rich environment of water (approximately 10 mM), this transformation was not limited by calcium abundance. However, the calcium-poor conditions on land created an evolutionary pressure to tightly regulate the concentration of calcium in intra- and extracellular compartments of the body. Since the largest amounts of calcium are stored in bones, they serve as reservoirs to balance variations in the supply of the mineral by diet. In this process, vitamin D, as well as the peptide hormone PTH (parathyroid hormone), took the lead role. For example, the calcium channel TRPV6 (transient receptor potential cation channel subfamily V member 6), as well as the calcium-binding proteins CALB1 (calbindin 1) and CALB2 are encoded by vitamin D target genes [57].

Vitamin D regulates the activity of bone-resorbing osteoclasts by the cytokine RANKL, which is encoded by the vitamin D target gene *TNFSF11* (TNF superfamily member 11) [58]. Moreover, also bone mineralization is controlled by proteins encoded by vitamin D target genes, such as SPP1 (osteopontin) and BGLAP (bone gamma-carboxyglutamate protein, also called osteocalcin). Bone remodeling, i.e., the resorption of extracellular matrix by osteoclasts as well as bone formation by osteoblasts, requires, like immune functions, also a lot of energy. [59]. Thus, bone remodeling and immunity are connected via their dependency on energy metabolism [60]. Moreover, hematopoietic stem cells find in the interior of large bones, the bone marrow, a niche, i.e., a place where proliferating immune cells are effectively shielded from radiation and, in parallel, supported by calcium. Interestingly, already in bone fishes like zebrafish (*Danio rerio*) vitamin D regulates hematopoietic cell growth during embryogenesis [61]. Thus, the close connection of calcium homeostasis and bone remodeling to immunity, i.e., the co-evolution of both systems, illustrates why vitamin D shifted into this additional task.

Taken together, vitamin D evolved from one of the multiple factors controlling (energy) metabolism and immunity to a dominant regulator of calcium homeostasis and bone remodeling. This explains why bone malformations were observed as the first symptom of vitamin D deficiency.

## 5. How Does the Evolution of *Homo sapiens* Relate to Vitamin D?

The anatomically modern human (*Homo sapiens*) evolved just some 300,000 years ago in East Africa [62] and spread then over the whole continent. In order to protect from sunburn and skin cancer induced by the intensive equatorial sun, the skin of these humans was profoundly pigmented [63,64]. Despite dark pigmentation, there was and still is sufficient vitamin D_3_ synthesis [65]. Just some 75,000 years ago, modern humans started to migrate to Asia and from there to Oceania, Europe and the Americas [66,67] (Figure 2). In Europe and in northern parts of Asia they experienced cold winter climates that let them cover their skin by clothes. In addition, the intensity of UV-B is at higher northern latitudes far lower, and in winter, for a few months, the radiation does not reach the surface [68]. Both clothing and northern latitude reduced the amount of vitamin D_3_ produced in the skin and could cause vitamin D deficiency. The medical consequences of vitamin D deficiency, bone malformations and reduced potency of the immune system, may have created an evolutionary pressure that could have pushed for a reduced skin pigmentation [69], in order to explain today’s North–South gradient in skin color [70].

**Figure 3:**
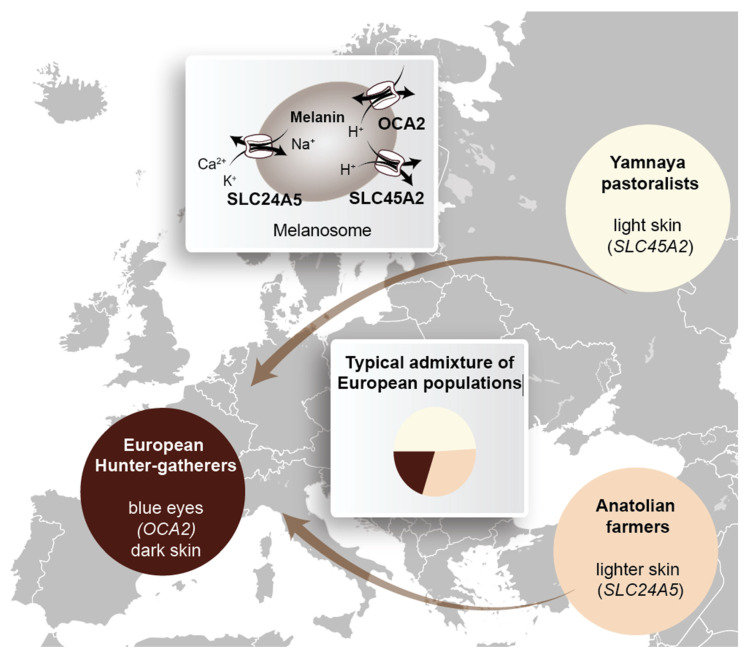
Schematic representation of the admixture of the European population. All European populations derived from European hunter–gatherers, Anatolian farmers and Yamnaya pastoralists [84]. A pie chart indicates the typical admixture of the founding populations (**center**). Melanin is produced in melanosomes under the control of the proteins OCA2, SLC24A5 and SLC45A2 (**top**).

Skin pigmentation depends on the load of keratinocytes with melanosomes [71], which are melanin-loaded organelles that origin from melanocytes [72] (Figure 3). The UV absorbing pigment melanin is produced via oxidation and polymerization of the aromatic amino acid tyrosine. The brown/black eumelanin is the most common form of melanin, while pheomelanin is yellow/red [73]. The difference in skin pigmentation of human populations and individuals (as well as that of their eyes and hair) primarily depends on SNPs (single nucleotide polymorphisms) in genes encoding for key proteins in melanogenesis [72]. The most relevant SNPs are those related to the genes *SLC24A5* (solute carrier family 24 member 5), *SLC45A2* and *OCA2* (OCA2 melanosomal transmembrane protein) [67,74] that encode for a potassium-dependent sodium/calcium exchanger, an ion transporter and a pH regulator in melanosomes, respectively [72]. Thus, the loss of function of these key proteins in melanin production leads to reduced skin pigmentation.

Modern humans arrived in Europe some 42,000 years ago [75,76] with dark skin like their African ancestors, but many of them had blue eyes due to variations of their *OCA2* gene [75,77] (Figure 3). By interbreeding, they outnumbered the ancestral Neanderthal hominins, which had lived in Europe already for some 400,000 years [77,78,79]. In net effect, today’s Europeans have, on average, 2.3% Neanderthal DNA in their genomes [80]. These hunter–gatherers lived first in ice-free southwestern Europe [81] and started some 11–12,000 years ago to colonize also northern Europe [75]. Based on archeogenomic data the evolution and timing of trait changes within European populations had been discovered [82] (Figure 3). First, some 8400 to 6000 years ago, people from northwestern Anatolia spread over southern Europe. These Anatolian farmers started the Neolithic revolution in Europe by introducing the concept of agriculture, i.e., the domestication of animal and plant species, to the hunter-gatherers. Moreover, by interbreeding with the indigenous European population, the Anatolian farmers also brought them their *SLC24A5* gene variant for lighter skin. In a second wave, some 5000 years ago, Yamnaya pastoralists from the Eurasian steppe arrived in Europe and settled preferentially in the North (Figure 3). They introduced the horse, the wheel, their Indo-European languages as well as lighter skin due to SNPs in their *SLC45A2* and *SLC24A5* genes to the preexisting European populations [83,84,85]. Thus, the relative admixture of the hunter-gatherers, Anatolian farmers and Yamnaya pastoralists explains the variation in skin color (as well as many other traits) of present Europeans.

Archeogenomic data demonstrated that *Homo sapiens* hunter-gatherer populations lived in Europe with dark skin for more than 30,000 years. Did they suffer from consequences of vitamin D deficiency? With the exception of people living an urban lifestyle already some 2000 years ago in the Roman empire [86], older bone samples do not show signs of malformation. Explanations could be a dominant outdoor lifestyle in southern Europe (the rest of Europe was covered by ice) or, in part, vitamin D_3_ supplementation via a marine-based diet for populations living close to the coast. However, the most dominant effects were SNPs in the regulatory regions of the *DHCR7* (7-dehydrocholesterol reductase) gene, which reduced its expression, and by this, its enzymatic activity [82,87]. The resulting increased concentrations of 7-dehydrocholesterol in the skin led then to a more efficient synthesis of vitamin D_3_ (Figure 1). Genome-wide association studies (GWAS) confirmed that the vitamin D status (as measured by 25(OH)D_3_ serum levels) significantly depends on SNPs of the *DHCR7* gene [88]. Furthermore, other GWAS demonstrated the dependence of the vitamin D status on genes related to vitamin D endocrinology, such as *CYP2R1*, *CYP24A1* and *GC*, but not to skin color [89,90]. This suggests that at least in (western) Europe, skin lightening did not happen due to an evolutionary pressure caused by vitamin D deficiency but by interbreeding with populations from northwestern Anatolia and the northern Caucasus. Thus, the light skin color of today’s Europeans is primarily based on the migration of populations from western Asia and the Near East to Europe [75]. Nevertheless, concerning their vitamin D status and their ability to populate also northern regions, the European populations benefitted from skin lightening.

## 6. How Vitamin D Became a Vitamin?

With the exception of highly developed societies, such as in the Roman empire, in the past, humans exposed larger percentages of their skin to the sun for a far longer part of the day than nowadays. In their evolutionary origin in East Africa, humans were every day around the year exposed to extensive UV-B radiation, which induced sufficient vitamin D_3_ synthesis. Therefore, over a period of more than 200,000 years, they got used to a constantly high vitamin D status of 100 nM 25(OH)D_3_ or more [65]. However, within the last 50–75,000 years, the migration toward regions with a latitude above 37 °N let them experience seasonal changes in sun exposure and periods of the year when vitamin D_3_ cannot be produced endogenously. Furthermore, as a result of the industrial revolution, humans adapted to an urban lifestyle with predominant indoor work and activity. Both conditions, vitamin D winters and indoor preferences, often led to vitamin D deficiency in industrialized countries. For example, in England in the 19th century, rickets, also called the “English disease”, was a very common disorder in children [91,92]. Moreover, also the severity of tuberculosis was and still is significantly increased in vitamin D deficient individuals [93]. Thus, not evolution but human migration and lifestyle changes made vitamin D_3_ a vitamin.

## 7. Conclusions

Vitamin D_2_ and vitamin D_3_ started their “career” more than a billion years ago as side products of sterol biosynthesis, in fungi, some plants and animals that scavenge UV-B radiation. Just half a billion years later, the endocrinology of vitamin D evolved and the biologically active form of vitamin D_3_, 1,25(OH)_2_D_3_, became a hormone. Via its high-affinity receptor VDR 1,25(OH)_2_D_3_ regulates genes that are involved in detoxification, energy metabolism, immunity and calcium homeostasis. With this pleiotropic functional profile vitamin D is an important contributor to organismal homeostasis and health. This explains why rather recently (from an evolutionary perspective) changes in human lifestyle caused a reduced endogenous vitamin D_3_ production. Since, in parallel, the majority of human populations are not adapted to a marine-based diet [94], i.e., their average dietary intake of vitamin D is low, vitamin D deficiency became a common problem. Worldwide, vitamin D deficiency affects more than a billion people [95] and causes health problems, such as bone malformations and a decreased potency of the immune system.
